# Radiative heat transfer exceeding the blackbody limit between macroscale planar surfaces separated by a nanosize vacuum gap

**DOI:** 10.1038/ncomms12900

**Published:** 2016-09-29

**Authors:** Michael P. Bernardi, Daniel Milovich, Mathieu Francoeur

**Affiliations:** 1 Radiative Energy Transfer Laboratory, Department of Mechanical Engineering, University of Utah, Salt Lake City, Utah 84112, USA

## Abstract

Using Rytov's fluctuational electrodynamics framework, Polder and Van Hove predicted that radiative heat transfer between planar surfaces separated by a vacuum gap smaller than the thermal wavelength exceeds the blackbody limit due to tunnelling of evanescent modes. This finding has led to the conceptualization of systems capitalizing on evanescent modes such as thermophotovoltaic converters and thermal rectifiers. Their development is, however, limited by the lack of devices enabling radiative transfer between macroscale planar surfaces separated by a nanosize vacuum gap. Here we measure radiative heat transfer for large temperature differences (∼120 K) using a custom-fabricated device in which the gap separating two 5 × 5 mm^2^ intrinsic silicon planar surfaces is modulated from 3,500 to 150 nm. A substantial enhancement over the blackbody limit by a factor of 8.4 is reported for a 150-nm-thick gap. Our device paves the way for the establishment of novel evanescent wave-based systems.

## Abstract (web summary)

Evanescent coupling between surfaces separated by a distance smaller than the thermal wavelength can lead to radiative heat transfer greater than the blackbody limit. Here, the authors demonstrate this between two macroscopic-scale surfaces, paving the way to harnessing the effect in thermal devices.

## Graphical abstract

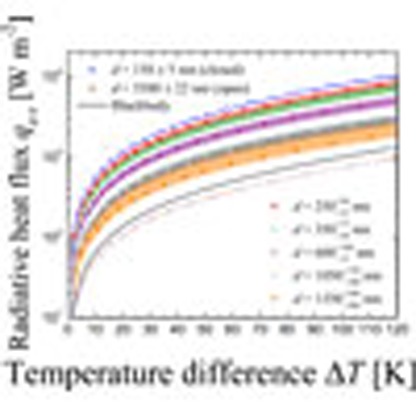

Radiation heat transfer exceeding the blackbody limit at nanosize separation gaps has been experimentally confirmed in the scanning probe-surface^1^,^2^,^3^, scanning probe-film^4^, microsphere-surface^5^,^6^,^7^,^8^, microsphere-film^9^ and microsphere-nanostructured surface^10^ configurations. Although the accuracy of fluctuational electrodynamics at sub-10 nm gaps has been questioned in the experiments of Kittel *et al*.^2^, the validity of this framework has been confirmed both experimentally^3^ and theoretically^11^ down to separation gaps of 2 and 1 nm, respectively. Additional work involving micro/nanostructures has also experimentally demonstrated the enhancement of thermal radiation in the near field^12^,^13^,^14^,^15^,^16^. The micro/nanosize surfaces involved in the aforementioned experiments, however, limit the amount of radiation that can be exchanged, such that these configurations cannot be readily applied to engineering systems such as thermophotovoltaic power generators^17^,^18^,^19^,^20^,^21^,^22^,^23^,^24^,^25^,^26^ and thermal rectifiers^27^,^28^,^29^,^30^,^31^. While the development of evanescent wave-based devices typically requires macroscale surfaces separated by a nanosize vacuum gap, experimental research on near-field radiative heat transfer between macroscale surfaces has mainly focused on relatively large, microsize separation gaps at cryogenic^32^,^33^ and room^34^,^35^,^36^,^37^ temperatures. Recently, Ito *et al*.^38^ measured radiative heat transfer between millimetre-size fused quartz surfaces separated by pillars, also made of fused quartz, at a separation gap of 500 nm. The results were twice that of fluctuational electrodynamics predictions because of excessive heat conduction through the pillars, which prevent the application of this configuration to engineering systems. Lim *et al*.^39^ measured a radiative heat transfer enhancement of 2.91 relative to the blackbody limit between two microstrips of doped silicon (Si) separated by a 400-nm-thick gap. Yet, significant heat transfer and radiation enhancement necessitate larger surfaces and a smaller separation gap, respectively.

The difficulty associated with maintaining a nanosize vacuum gap between macroscale planar surfaces is the main bottleneck, currently preventing the application of near-field thermal radiation to engineering systems^15^.

Here we address this bottleneck by measuring radiative heat transfer via a custom-fabricated device consisting of two planar 5 × 5 mm^2^ intrinsic Si surfaces separated by a gap that can be modulated from 3,500 nm down to 150 nm via a compliant membrane and mechanical actuation. This device enables probing radiative heat transfer between macroscale surfaces for large temperature differences (Δ*T*∼120 K) in multiple regimes, including those dominated by either propagating or evanescent modes. An excellent agreement between experimental results and fluctuational electrodynamics predictions^40^,^41^ is obtained, and a radiative transfer enhancement of 8.4 relative to the blackbody limit is measured for a separation gap of 150 nm. The potential application of our device architecture to thermophotovoltaic power generation is also discussed.

## Results

### Experimental procedure

**Figure 1:**
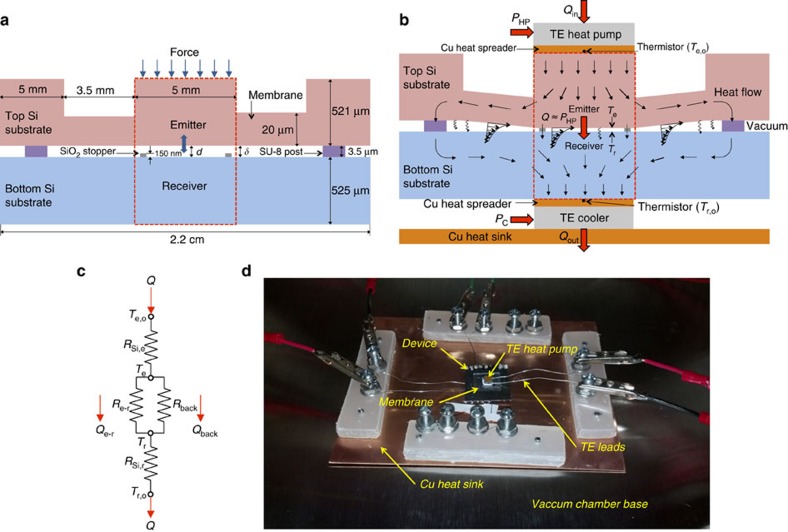
Near-field radiative heat transfer device. (**a**) Device in the open position, where the emitter–receiver portion is identified by a dashed box. It consists of two silicon (Si) substrates separated by 3.5-μm-tall SU-8 posts. The gap between the emitter and the receiver, *d*, can be modulated by applying a force causing the membrane to flex. The minimum gap *d* is 150 nm and corresponds to the height of the silicon dioxide (SiO_2_) stoppers. The gap *d* is uniform under an applied force, while the gap outside the emitter–receiver region, *δ*, is non-uniform when a force is applied to the device. The 1-μm-thick buried SiO_2_ layer in the top Si substrate is not shown. (**b**) Heat flow through the device in the closed position. The temperature difference is maintained by a TE heat pump and a TE cooler supplying power of *P*_HP_ and *P*_C_, respectively. *P*_HP_ is approximately equal to the heat rate through the device, *Q*, since the heat rate *Q*_in_ due to thermal emission by the surroundings is negligible with respect to *P*_HP_. Heat transfer between the top and bottom Si substrates occurs via radiation within and outside the emitter–receiver region and by conduction through the SU-8 posts and SiO_2_ stoppers when the device is in the closed position. The temperatures *T*_e,o_ and *T*_r,o_ are measured by thermistors and are approximately equal to *T*_e_ and *T*_r_, respectively. The device is mounted on a copper (Cu) heat sink dissipating heat, *Q*_out_, to the base of the vacuum chamber. (**c**) Equivalent thermal circuit of the device. *R*_Si,e_ and *R*_Si,r_ are the resistances of the Si emitter and receiver, *R*_e–r_ is the resistance due to radiation between the emitter and receiver, *R*_back_ is the resistance due to background heat transfer that includes radiation outside the emitter–receiver region as well as conduction through the SU-8 posts and SiO_2_ stoppers. The heat rate flowing through the device, *Q*, is the sum of heat rates due to radiation between the emitter and receiver, *Q*_e–r_, and background heat transfer, *Q*_back_. (**d**) Photograph of the near-field radiative heat transfer device in the vacuum chamber.

The near-field radiative heat transfer device, shown in Fig. 1, was manufactured using standard microfabrication techniques, as detailed in Supplementary Note 1 and Supplementary Fig. 1. The device consists of two 2.2 × 2.2 cm^2^ Si substrates separated by four rigid, 3.5-μm-tall SU-8 posts with a diameter of 250 μm. The bottom substrate was fabricated from a 525-μm-thick intrinsic Si wafer, while the top substrate was manufactured from a 521-μm-thick Si-on-insulator wafer with a 1-μm-thick buried silicon dioxide (SiO_2_) layer. The surface roughness of the Si substrates was less than 1.2 nm as measured with a Zygo NewView optical profilometer. A 501-μm-deep, 3.5-mm-wide trench was etched on the backside of the top Si substrate using deep-reactive ion etching and a buffered oxide etch solution. This resulted in a 20-μm-thick compliant Si membrane, allowing the 5 × 5 mm^2^ emitter to move relative to the receiver under an applied force (the emitter–receiver portion of the device is identified by a dashed box in Fig. 1a,b). To avoid contact between the emitter and the receiver, four SiO_2_ stoppers with a diameter of 5 μm and height of 150 nm were fabricated on the lower Si substrate, thus fixing the minimum separation gap *d* to 150 nm. The two Si substrates were precisely aligned and bonded in an EVG 520 IS wafer bonder. Testing of the device was conducted in a vacuum chamber (*P*≈10^−4^ Pa) located in a class 1,000 clean room tent.

Heat transfer measurements were performed using the configuration shown in Fig. 1b,c. The temperature difference was maintained via thermoelectric (TE) modules (Custom Thermoelectric, 00701-9B30-22RU4) acting as a heat pump and cooler on the emitter and receiver sides, respectively. These TE modules enabled a maximum temperature difference of ∼120 K between the emitter and the receiver. The TE heat pump was mounted on a 500-μm-thick copper (Cu) heat spreader located on the 5 × 5 mm^2^ Si emitter. The temperature of the outer surface of the emitter *T*_e,o_ was measured via a thermistor (Selco, LSMC700A010KD002) embedded in the Cu heat spreader. The outer surface of the receiver was maintained at a temperature *T*_r,o_ of 300 K, monitored via a thermistor, by the TE cooler. The receiver and TE cooler were separated by a 500-μm-thick Cu heat spreader. The entire device was placed on a Cu heat sink mounted to the base of the vacuum chamber (see Fig. 1d) and all contact resistances were minimized using thermal grease (Arctic Silver Ceramique 2). Heat was supplied to the device at a rate *Q*, which is the sum of the heat rate into (*Q*_in_) and supplied by (*P*_HP_) the TE heat pump. Since the device is in a vacuum, *Q*_in_ is solely due to thermal emission by the stainless steel walls and aluminium door of the vacuum chamber near ambient temperature and is thus much smaller than *P*_HP_, such that *Q*≈*P*_HP_. The heat supplied by the TE heat pump *Q*, partially spreading outside the emitter–receiver portion of the device, is divided into two contributions, namely radiation heat transfer at a separation gap *d* between the emitter and receiver *Q*_e–r_, and the background heat transfer *Q*_back_. The background heat rate *Q*_back_ includes radiation outside the emitter–receiver portion of the device at a separation gap *δ*, conduction through the SU-8 posts and conduction through the SiO_2_ stoppers when the device is in the closed position. Note that the thermal resistance associated with the separation gap (for example, 462.4 K W^−1^ for *d*=150 nm, *T*_e,o_=420 K, *T*_r,o_=300 K) is much larger than the thermal resistances of the emitter (0.192 K W^−1^) and receiver (0.162 K W^−1^), such that the measured temperatures are approximately equal to temperatures of the inner surfaces of the emitter (*T*_e_) and receiver (*T*_r_), adjacent to the vacuum gap. The experimental procedure was validated by measuring conduction through a 1.1-mm-thick layer of borosilicate glass. In addition, using a technique similar to that of Hu *et al*.^35^, radiation transfer was measured between 5 × 5 mm^2^ planar Si surfaces separated by vacuum gaps of 500 and 200 nm maintained by low thermal conductivity (0.18 W m^−1^ K^−1^) polystyrene spherical particles. The validation procedure is detailed in Supplementary Note 2, while the conduction and radiation validation results are provided in Supplementary Figs 2 and 3, respectively.

### Heat transfer measurements

**Figure 2:**
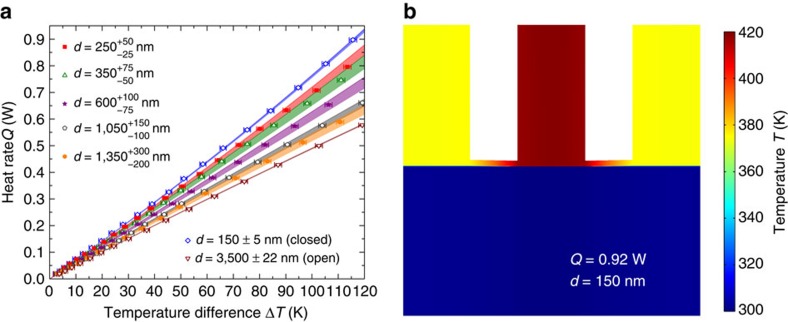
Gap- and temperature-dependent heat rate. (**a**) Heat rate, *Q*, as a function of the temperature difference between the emitter and receiver, Δ*T*, for various separation gaps, *d*. In all cases, the temperature of the receiver, *T*_r_, is fixed at 300 K. The symbols show unprocessed experimental data, while the coloured bands are numerical simulations obtained from the coupled fluctuational electrodynamics-COMSOL Multiphysics comprehensive model. The gap sizes *d* in the open and closed positions are known, with some small uncertainty, from the manufacturing of the device and the associated measured heat rates are in good agreement with numerical predictions. It was not possible to measure directly the intermediate gap sizes, such that they were estimated from the comprehensive heat transfer model. (**b**) Simulated temperature distribution in the device via the comprehensive model for an input heat rate *Q* of 0.92 W, a separation gap *d* of 150 nm and a fixed receiver temperature *T*_r_ of 300 K resulting in an emitter temperature of 420 K. Heat spreading outside the emitter portion of the device results in background heat transfer *Q*_back_.

The heat rate supplied by the TE heat pump as a function of the measured temperature difference Δ*T* between the emitter and receiver was compared against numerical predictions. Unprocessed experimental heat rates *Q* that include radiation heat transfer between the emitter and receiver *Q*_e–r_ as well as the background heat transfer *Q*_back_ are shown in Fig. 2a for temperature differences Δ*T* (=*T*_e_–*T*_r_) up to 120 K. The separation gap *d* between the emitter and receiver was modulated by using calibrated masses ranging from 0.9 to 5 g, as detailed in Supplementary Note 3. The numerical predictions were obtained via a coupled fluctuational electrodynamics-COMSOL Multiphysics comprehensive heat transfer model of the device taking into account radiation between the emitter and receiver, heat transfer by radiation outside the emitter–receiver region and conduction through the SiO_2_ stoppers and SU-8 posts. The details of the comprehensive model are provided in the Methods Section. Figure 2b shows the temperature distribution in the device obtained from the model for a heat rate *Q* of 0.92 W and a separation gap *d* of 150 nm. The agreement between experimental data and numerical predictions when the device is in the open (*d*=3,500±22 nm) and closed (*d*=150±5 nm) position is remarkable. The uncertainty associated with these gap sizes, identified as coloured bands in Fig. 2a, was determined experimentally by measuring the variation of the height of the SiO_2_ and SU-8 layers used to create the stoppers and the posts, respectively. Using nominal gap values of 3,500 and 150 nm in the numerical simulations, a maximum relative difference between experiments and predictions of 9.1% is obtained for a 3,500-nm-thick gap and Δ*T*=15.5 K, while a minimum relative difference of less than 0.1% is achieved for a 150-nm-thick gap and Δ*T*=84.2 K. In addition, for all cases presented in Fig. 2a, radiation largely dominates heat transfer through the device. According to the model, the portion of the heat rate because of conduction reaches a maximum of 11.7% when the gap size is 3,500 nm and the temperature difference is 3.8 K. For the case when the device is in the closed position (*d*=150 nm) and the temperature difference is 115.6 K, the portion of the total heat rate due to conduction is at a minimum of 6.6%. As explained in Supplementary Note 3 and Supplementary Fig. 4, it was not possible to determine exactly the intermediate gap sizes between the open and closed positions. As the force applied on the device was increased, it was observed that the heat rate *Q* increased, because of a larger proportion of evanescent modes contributing to heat transfer, until the device was in the closed position. Intermediate gap sizes, shown in Fig. 2a, were estimated using the comprehensive model in combination with the measured heat rates and temperatures (see Methods section).

**Figure 3:**
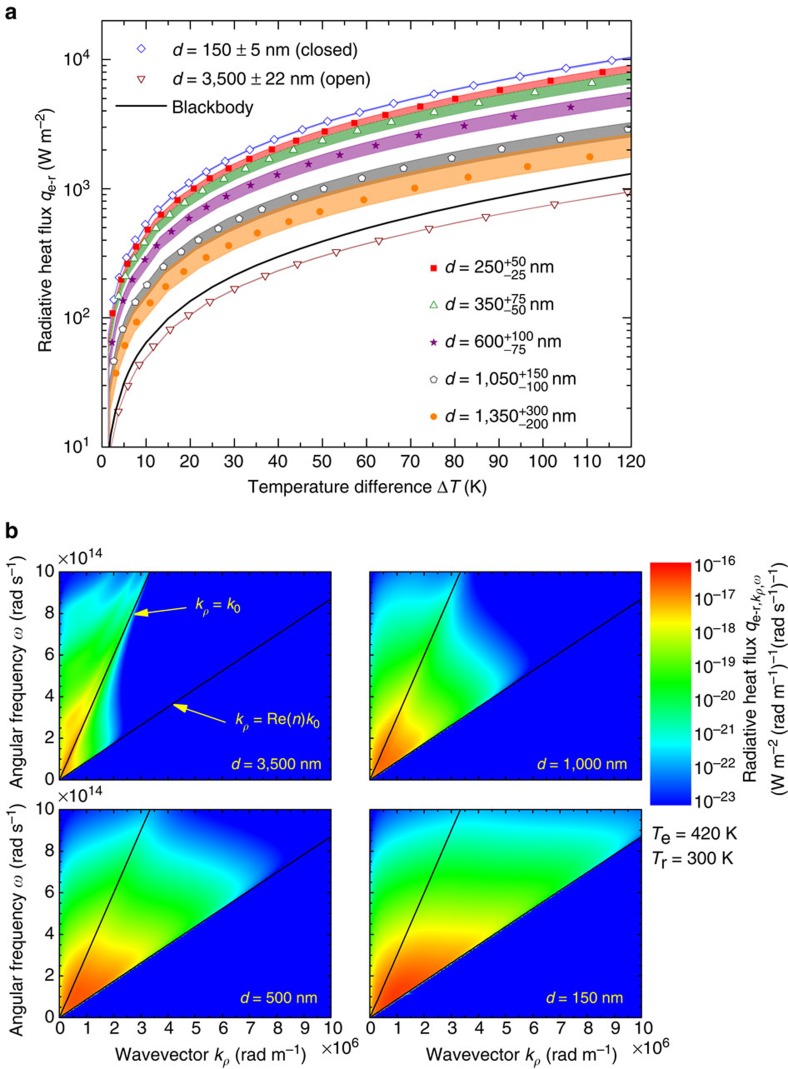
Gap- and temperature-dependent radiative heat flux between the emitter and receiver. (**a**) The symbols show the experimental heat flux between the 5 × 5 mm^2^ emitter and receiver, *q*_e–r_, where the background heat transfer has been subtracted. The coloured bands are fluctuational electrodynamics predictions. In the closed position (*d*=150 nm) and for a temperature difference Δ*T* of 115.6 K, the experimental radiative heat flux exceeds the blackbody predictions by a factor of 8.4. In the open position (*d*=3,500 nm), the measured radiative heat flux is below the blackbody predictions due to a modest contribution from evanescent modes. (**b**) Calculated dispersion relations showing the radiative heat flux, *q*_e–r,*k*_*ρ*_,*ω*_, per unit parallel wavevector *k*_*ρ*_ and angular frequency *ω* for gaps of 3,500, 1,000, 500 and 150 nm, and emitter (*T*_e_) and receiver (*T*_r_) temperatures of 420 and 300 K, respectively. The region where *k*_*ρ*_ is smaller than the magnitude of the wavevector in vacuum, *k*_0_, corresponds to modes that are propagating in the vacuum gap. The zone defined by *k*_0_<*k*_*ρ*_<Re(*n*)*k*_0_, where *n* is the refractive index of silicon (Si), describes frustrated modes that are propagating in Si but evanescent in the vacuum gap. Radiation enhancement in the near field for the case of intrinsic Si is solely due to these frustrated modes that have an increasing contribution to heat transfer as the separation gap *d* decreases.

The radiative heat flux between the 5 × 5 mm^2^ emitter and receiver without the background heat rate is shown in Fig. 3a as a function of the temperature difference. The agreement between experimental data and fluctuational electrodynamics predictions in the open and closed positions, for which the gap sizes were measured experimentally, is excellent. A maximum radiation transfer enhancement over the blackbody limit by a factor of 8.4 was measured for a gap size of 150 nm and a temperature difference of 115.6 K. This constitutes the largest value recorded between two macroscale planar surfaces at non-cryogenic temperatures. The mechanism responsible for this enhancement can be understood by inspecting the dispersion relations shown in Fig. 3b, where the heat flux is plotted as a function of the angular frequency *ω* and parallel wavevector *k*_*ρ*_ for gap sizes of 3,500, 1,000, 500 and 150 nm, and a temperature difference of 120 K. Modes that are propagating in both Si and vacuum are contained within the region *k*_*ρ*_<*k*_0_ (=*ω*/*c*_0_), where *k*_0_ is the magnitude of the wavevector in vacuum. Planck's theory of heat radiation solely accounts for these modes. Frustrated modes, propagating in Si and evanescent in vacuum, are characterized by parallel wavevectors *k*_0_<*k*_*ρ*_<Re(*n*)*k*_0_, where *n* is the refractive index of Si. Surface modes are evanescent in both Si and vacuum and are described by *k*_*ρ*_>Re(*n*)*k*_0_. The dispersion relations show clearly that the enhancement of radiative heat transfer is solely due to the additional contribution of frustrated modes in the near field, as intrinsic Si does not support surface modes such as surface phonon–polaritons or surface plasmon–polaritons. Here with a single device, we measured radiative heat transfer in various regimes, including those dominated by either propagating or evanescent modes. Indeed, although heat transfer via frustrated modes occurs at a gap of 3,500 nm, their contribution is insufficient to exceed the blackbody predictions (∼68% of the heat flux is due to propagating modes). Conversely, radiation heat transfer is largely dominated by frustrated modes for a 150-nm-thick gap and accounts for ∼88% of the heat flux between the emitter and the receiver.

## Discussion

Near-field thermal radiation research is motivated by potential applications to energy conversion, heat flow management, imaging and micro/nanomanufacturing^17^,^21^,^31^,^42^,^43^,^44^,^45^,^46^,^47^. Among those, thermophotovoltaic power generation capitalizing on evanescent modes is a promising application of our device, as this technology requires both large surfaces and considerable near-field enhancement. For instance, when considering both the near-field enhancement and the size of the surfaces, our device provides a radiative heat rate more than one order of magnitude larger than in ref. 39. In addition, conductive heat losses in our device, negatively having an impact on thermophotovoltaic power generation^22^,^25^,^48^, are minimized to 6.6% of the total heat rate when the near-field enhancement is maximum. For an emitter temperature of 420 K, the photon energy at which thermal emission is maximum is ∼0.18 eV, such that our device could operate as a thermophotovoltaic power generator by replacing the receiver by a cell made of indium antimonide (InSb) having an absorption bandgap energy of 0.17 eV (ref. 26). In the radiative limit^23^, we estimated that the output power density and conversion efficiency of a thermophotovoltaic power generator made of intrinsic Si and InSb maintained at 420 and 300 K, respectively, were 400 W m^−2^ and 2.9% for a 150-nm-thick gap. This implies that a 5 × 5 mm^2^ surface area would produce an electrical power of ∼10 mW, a value that could be increased by using an array of devices. In comparison, a similar thermophotovoltaic system operating in the far-field regime with a blackbody source would lead to a significantly lower output power density of 33.4 W m^−2^ and the same conversion efficiency of 2.9%. A thermophotovoltaic device operating with an emitter temperature of 420 K could potentially be used for recycling waste heat in electronic devices such as solar cells, where output power density is more important than conversion efficiency^48^. In an actual thermophotovoltaic power generator, one must also consider the impacts of electrical and thermal losses on system performance. This was done by Bernardi *et al*.^25^, where the results showed that, despite its broadband near-field enhancement, an emitter such as intrinsic Si supporting strictly propagating and frustrated modes is more beneficial to the performance of evanescent wave-based thermophotovoltaic power generators than a radiatively optimized emitter supporting surface modes.

In summary, using a custom-fabricated device made of two 5 × 5 mm^2^ intrinsic Si surfaces separated by a tunable vacuum gap, we measured a maximum radiation transfer enhancement over the blackbody limit by a factor of 8.4 for a 150-nm-thick gap and a temperature difference of 115.6 K while minimizing heat conduction. Our near-field radiative heat transfer device, capable of delivering considerable radiation heat rates because of the large near-field enhancement and surface size, paves the way to the development of thermophotovoltaic systems converting evanescent modes into electrical power.

## Methods

### Experimental uncertainty analysis

The uncertainty associated with the experimental data is because of the temperature and heat rate measurements. For the temperature, the uncertainty stems from the ohmmeter (BK Precision, 889B) used to measure the resistance of the thermistors as well as the thermistors themselves. The error in the resistance measurement is given by the manufacturer specifications as ±(0.2%+0.1 Ω) within the range from 100 to 1,000 Ω, and ±(0.1%+1.0 Ω) for the range of 1–10 kΩ. The uncertainty introduced by the thermistors is a function of temperature and the thermistors' change of resistance with temperature. A resistance reading of 9,225 Ω corresponds to a temperature of 300±0.46 K while a resistance reading of 185.8 Ω corresponds to a temperature of 420±0.85 K. Combining the uncertainties introduced by the ohmmeter and the thermistors results in an overall uncertainty of ±0.48 K at 300 K and ±0.89 K at 420 K.

The uncertainty associated with the heat rate is introduced by the power supply (BK Precision, 9121 A) connected to the TE heat pump. The uncertainty in the supplied current is ±(0.05%+2 mA) and the uncertainty in the supplied voltage is ±(0.02%+3 mV). In addition, the resistance associated with the wires needs to be accounted for since it induces a small amount of power dissipation. A four-wire sensing technique was employed to account for the resistance of the wires leading up to the device. The resistance associated with the TE leads (see Fig. 1d) was measured to be 0.08±0.9 mΩ. For example, when 1.2 A±2.6 mA and 0.84 V±3.2 mV is provided by the power supply (1.01 W±6.0 mW), the power dissipated in the TE leads is 115.2±1.8 mW. The power supplied to the TE heat pump, which is equivalent to the total heat rate, is then determined to be 894.8±7.8 mW. These uncertainties are plotted as error bars in Fig. 2a.

### Computational model

Near-field radiative heat transfer was modelled using fluctuational electrodynamics^40^. The net radiative heat flux because of propagating and evanescent waves in the emitter–receiver portion of the device was calculated as follows^41^:


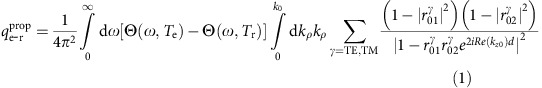



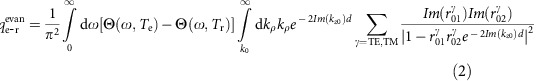


where the subscripts 0, 1 and 2, respectively, refer to the vacuum, the top Si substrate and bottom Si substrate, TE and TM refer to transverse electric and transverse magnetic polarizations, Θ(*ω*,*T*) is the mean energy of an electromagnetic state calculated as 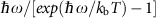, *k*_*ρ*_ and *k*_*z*_ are the components of the wavevector parallel and perpendicular to the surface of the layers, while 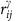 is the Fresnel reflection coefficient at the interface of media *i* and *j* in polarization state *γ*. Outside the emitter–receiver portion of the device, equations (1) and (2) were modified to account for the fact that the 20-μm-thick membrane was optically thin. As such, the background-radiative heat flux due to propagating and evanescent waves was calculated as^49^:


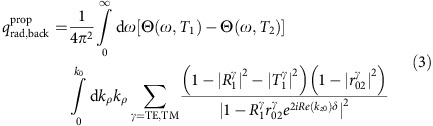



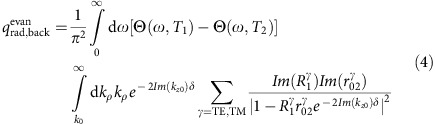


where 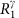 and 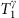 are the reflection and transmission coefficients of layer 1 in polarization state *γ*. These coefficients are calculated as 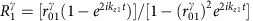 and 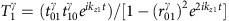, where *t* is the thickness of layer 1, while 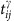 is the Fresnel transmission coefficient at the interface of media *i* and *j* in polarization state *γ*. The net radiative heat flux used for producing the numerical results shown in Fig. 2a,b was obtained by summing equations (1) and (2) in the emitter–receiver region, and by summing equations (3) and (4) outside the emitter–receiver region. The net radiative heat flux in the emitter–receiver portion of the device shown in Fig. 3a was calculated by summing equations (1) and (2). The dispersion relations in Fig. 3b were generated by solving equations (1) and (2) per unit angular frequency *ω* and per unit parallel wavevector *k*_*ρ*_. Note that the dielectric function of intrinsic Si was assumed to be independent of temperature in the range of operation and was obtained by curve-fitting the experimental data from ref. 50.

Theoretical curves of heat rate *Q* as a function of the temperature difference Δ*T* between the emitter and receiver for a specific separation gap *d* were calculated using a coupled fluctuational electrodynamics-COMSOL Multiphysics comprehensive model to account for radiation transfer in the emitter–receiver region *Q*_e–r_ as well as the background heat transfer *Q*_back_. Near-field radiative heat transfer was included in COMSOL by defining a fictional material, in place of the vacuum gap, characterized by a local, temperature-dependent effective thermal conductivity. In the emitter–receiver portion of the device, the effective thermal conductivity was calculated as *k*_eff_=*qd*/Δ*T*, where *q* is the sum of equations (1) and (2). The effective thermal conductivity outside the emitter–receiver region was derived using *k*_eff_=*qδ*/Δ*T*, where *q* is the sum of equations (3) and (4), and by using the Derjaguin approximation^7^ to account for the variations of the separation distance *δ*, assumed to be linear, when the device was not in the open position. Heat conduction through the SU-8 posts and SiO_2_ stoppers was calculated using temperature-independent thermal conductivities of 0.2 and 1.3 W m^−1^ K^−1^ listed in refs 51, 52, respectively, while the temperature-dependent thermal conductivity provided in ref. 53 was used for intrinsic Si. A typical value for contact resistance of 2.5 × 10^−5^ K m^2^ W^−1^ was imposed between the Si and SiO_2_ stoppers as well as between the Si and SU-8 posts^54^. For a given gap *d* separating the emitter from the receiver, heat transfer simulations in the device were initiated by imposing the heat rate supplied by the TE heat pump *P*_HP_ (≈*Q*), while the TE cooler was modelled as a constant temperature boundary condition (300 K) at the bottom face of the receiver. For these conditions, the temperature distribution in the device, and thus the temperature difference Δ*T* between the emitter and receiver, was determined using an iterative method where the effective thermal conductivity of the vacuum gap and the thermal conductivity of Si were calculated at the updated temperature. Iterative computations were repeated until a maximum absolute temperature difference between two successive iterations less than 0.001 K was obtained everywhere in the spatial grid. These simulations were repeated for a series of heat inputs *Q* ranging from 0 to 1.4 W in increments of 0.04 W, thus allowing the generation of a theoretical heat rate *Q* as a function of the temperature difference Δ*T* between the emitter and receiver for a specific separation gap *d*. For instance, Fig. 2b shows the temperature distribution in the device for a heat input *Q* of 0.92 W and a separation gap *d* of 150 nm. For these conditions, the emitter reaches a uniform temperature of 420 K, but heat is also dissipated outside the emitter region in the top Si substrate. It can also be seen that the bottom Si substrate has a nearly uniform temperature of 300 K.

Validation of the coupled fluctuational electrodynamics-COMSOL Multiphysics model was performed by comparing numerical predictions against unprocessed experimental data measured when the device was in the open (*d*=3,500±22 nm) and closed (*d*=150±5 nm) positions. The uncertainty associated with these two gap sizes was used to calculate a theoretical band of heat rate *Q* as a function of the temperature difference Δ*T*. These bands, however, are fairly small because of the small uncertainty associated with these gap sizes, and are thus hardly visible in Fig. 2a.

### Estimation of intermediate separation gap sizes

There was no direct method for measuring the separation gap between the emitter and receiver, except when the device was in the open and closed positions. Since the coupled fluctuational electrodynamics-COMSOL Multiphysics model was in excellent agreement with the measured heat rate as a function of the temperature difference in both the open and closed positions (see Fig. 2a), intermediate separation gap sizes were estimated using the aforementioned model. Specifically, a nominal gap size *d* was determined by best fitting experimental data of nominal heat rate *Q* versus temperature difference Δ*T* with numerical predictions. The uncertainty associated with the estimated gaps, shown in coloured bands in Figs 2a and 3a, was derived by best fitting experimental data of maximum *Q* versus minimum Δ*T*, and minimum *Q* versus maximum Δ*T* with numerical predictions. These maximum and minimum values were determined from the uncertainty associated with the measured temperatures and heat rates.

Even if intermediate gap sizes were not determined from an independent measurement, the experimental results show clearly that heat transfer increases as the separation gap decreases because of an increasing contribution of evanescent modes. Heat transfer reaches saturation when the emitter comes into contact with the SiO_2_ stoppers. These observations are consistent with fluctuational electrodynamics predictions.

### Data availability

The data that support the findings of this study are available from the corresponding author upon request.

## Additional information

**How to cite this article:** Bernardi, M. P. *et al*. Radiative heat transfer exceeding the blackbody limit between macroscale planar surfaces separated by a nanosize vacuum gap. *Nat. Commun.* 7:12900 doi: 10.1038/ncomms12900 (2016).

## Supplementary Material

Supplementary Information Supplementary Figures 1-4, Supplementary Notes 1-3 and Supplementary References
