# Supplementary material for: Radiative heat transfer exceeding the blackbody limit between macroscale planar surfaces separated by a nanosize vacuum gap
Source: Nat Commun. 2016 Sep 29;7:12900. doi: 10.1038/ncomms12900 (PMC5056409; doi:10.1038/ncomms12900)
Supplement: Supplementary Information — Supplementary Figures 1-4, Supplementary Notes 1-3 and Supplementary References [file ncomms12900-s1.pdf]

SUPPLEMENTARY FIGURES

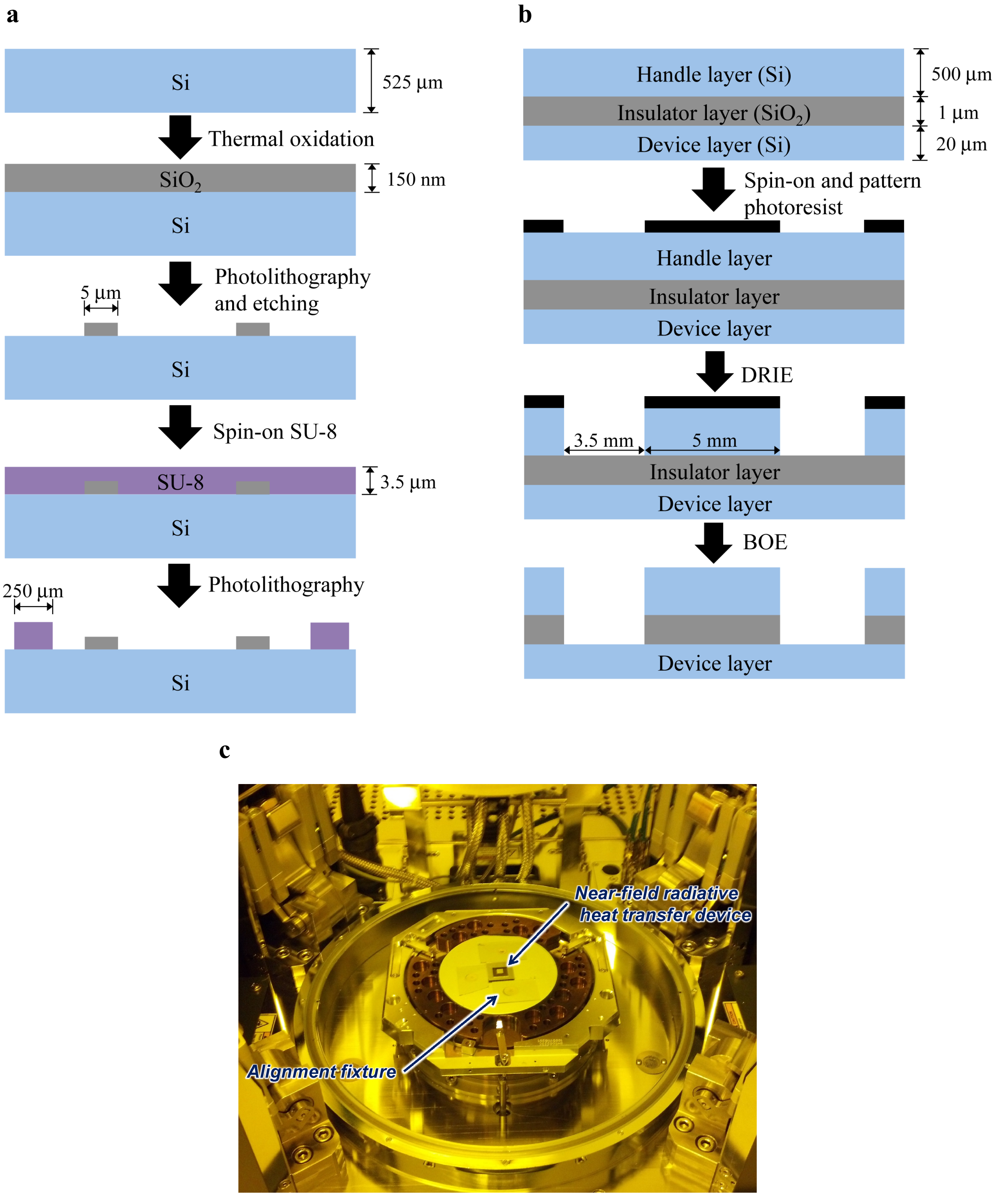

Supplementary Figure 1 | Fabrication of the near-field radiative heat transfer device. a, Main fabrication steps for the bottom Si substrate. **b,** Main fabrication steps for the top Si substrate using an SOI wafer. **c,** Interior of the EVG 520 IS wafer bonder. A custom-fabricated fixture was employed to ensure proper alignment of the top and bottom Si substrates.

a

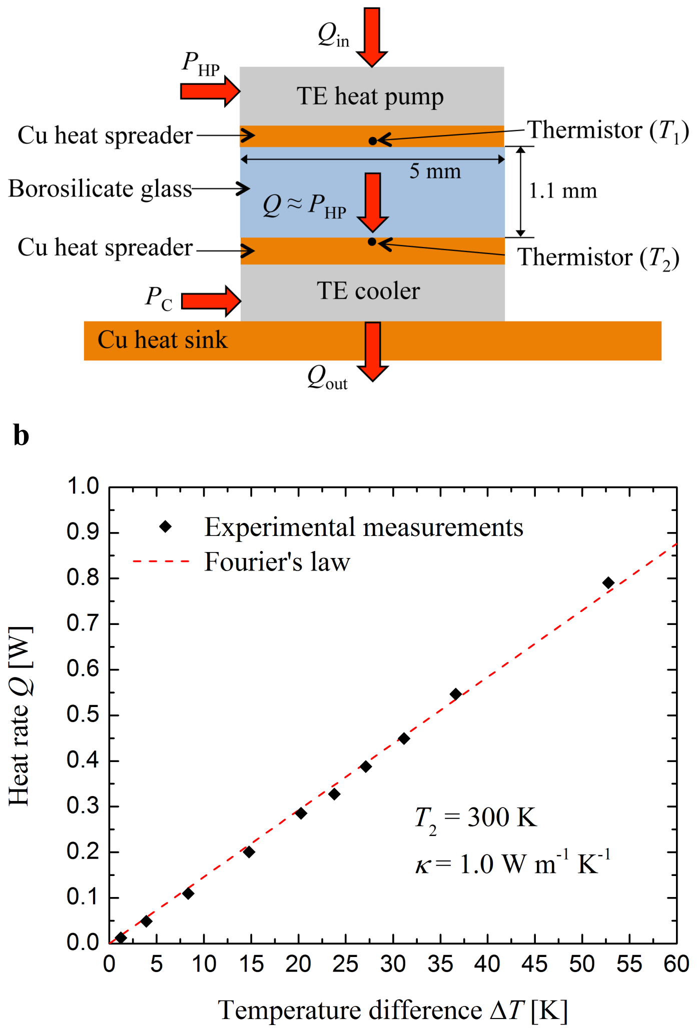

Supplementary Figure 2 | Calibration of the experimental setup by measuring conduction through borosilicate glass. a, Schematic of the experimental setup used to calibrate the measurement system for the case of conduction. It consists of a 1.1-mm-thick layer of borosilicate glass sandwiched between two copper (Cu) heat spreaders. The temperature difference is maintained by a thermoelectric (TE) heat pump and a TE cooler. The power supplied to the TE heat pump, P_{HP}, is approximately equal to the heat rate through the glass, Q. The temperatures on either side of the glass layer, T_1 and T_2, are measured using thermistors embedded in the Cu heat spreaders. **b,** Heat rate, Q, as a function of temperature difference, ΔT. The symbols indicate unprocessed experimental results while the dashed line correspond to Fourier's law using a thermal conductivity κ = 1.0 W m^{-1} K^{-1} for borosilicate glass.

a

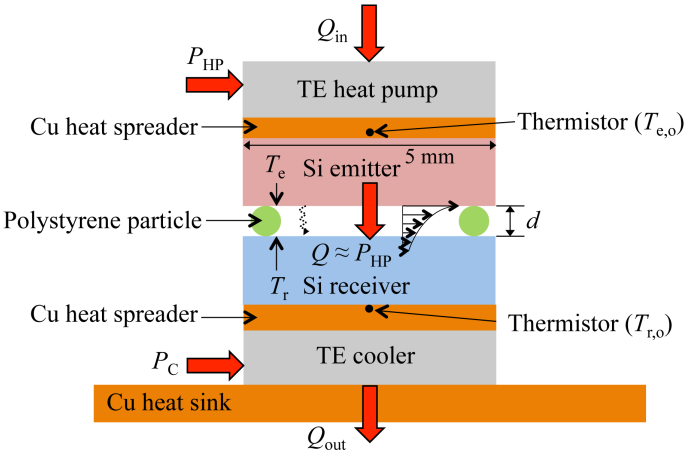

b

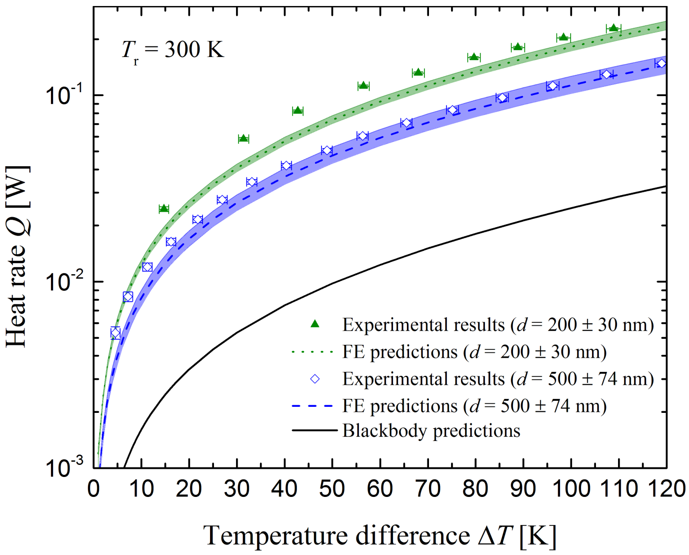

Supplementary Figure 3 | Calibration of the experimental setup by measuring radiation between Si surfaces separated by nanosize polystyrene spherical particles. a, Schematic of the experimental setup used to calibrate the measurement system for the case of near-field radiation. It consists of two silicon (Si) substrates separated by vacuum gap sizes of 500 nm and 200 nm using polystyrene spherical particles. The temperature difference between the emitter and receiver is maintained by a thermoelectric (TE) heat pump and a TE cooler. The power supplied to the TE heat pump, P_{HP}, is approximately equal to the heat rate, Q. The temperatures $T_{e,o}$ and $T_{r,o}$ are measured by thermistors and are approximately equal to T_e and T_r, respectively. **b,** Heat rate, Q, as a function of temperature difference between the emitter and receiver, ΔT. The symbols show unprocessed experimental measurements, while the colored bands are numerical simulations obtained from fluctuational electrodynamics (FE). Experimental results at a gap size of 500 nm exceed blackbody predictions by a factor of 4.6 at a temperature difference of 119.0 K. At a gap size of 200 nm, blackbody predictions are exceeded by a factor of 8.1 at a temperature difference of 108.9 K.

a

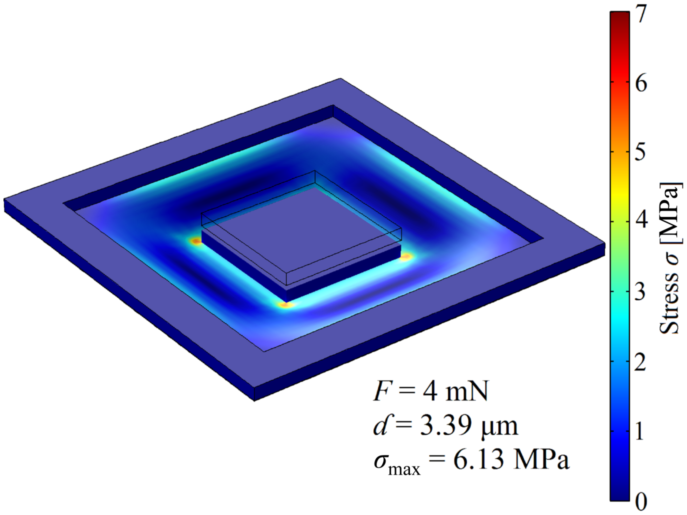

b

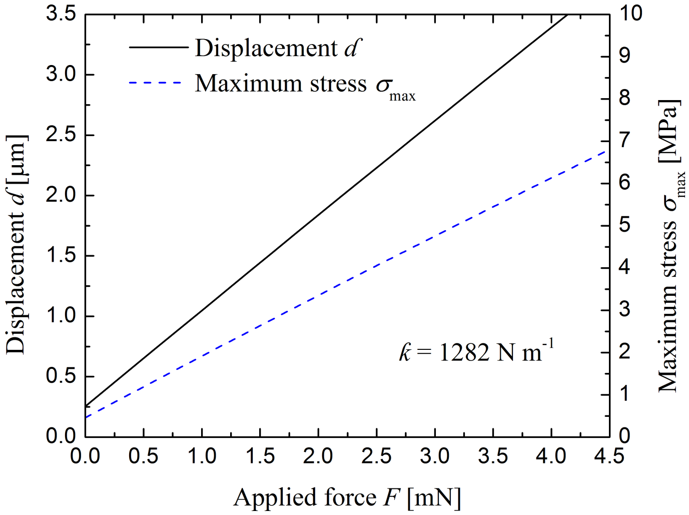

Supplementary Figure 4 | Modeling of the membrane. a, Stress distribution in the membrane when a force, *F*, of 4 mN is applied to the emitter. The maximum stress, σ_{max}, of 6.13 MPa occurring at the corners of the membrane is significantly smaller than the Si yield strength of 7 GPa. The model shows that the displacement, *d*, of the emitter relative to the receiver under an applied force of 4 mN is enough to bring the device in the closed position. **b,** Numerical predictions of the emitter displacement, *d*, and membrane maximum stress, σ_{max}, as a function of the applied force, *F*, for the 20-µm-thick, 3.5-mm-wide membrane. The resulting spring coefficient, *k*, is predicted to be 1282 N m^{-1}.

SUPPLEMENTARY NOTES

Supplementary Note 1. Device fabrication.

Fabrication of the bottom silicon (Si) substrate. The main steps required in fabricating the bottom Si substrate are shown in Supplementary Fig. 1a. Starting with a 525-µm-thick, 10-cm-diameter intrinsic Si wafer, a 150-nm-thick layer of silicon dioxide (SiO_2) was grown using thermal oxidation. Most of the SiO_2 layer was etched away using UV lithography and a buffered oxide etch (BOE) solution consisting of 25% HF:40% NH_4F leaving a set of four 5-µm-diameter stoppers per device. The purpose of these stoppers was to prevent the emitter from making contact with the receiver when the device was in the closed position. Next, a 3.5-µm-thick layer of SU-8 3005 negative photoresist was spun onto the wafer. A spin speed of 4500 rpm lasting 30 seconds was required to achieve the desired thickness. Using UV lithography, 250-µm-diameter areas were exposed and developed in order to create the posts (four per device) separating the top and bottom Si substrates. Six bottom Si substrates were created by dicing the wafer into 2.2 × 2.2 cm^2 sections.

Fabrication of the top Si substrate. The main steps required in fabricating the top Si substrate are shown in Supplementary Fig. 1b. Fabrication was performed on a 521-µm-thick, 10-cm-diameter Si-on-insulator (SOI) wafer. The SOI wafer consisted of a 20-µm-thick intrinsic Si device layer, a 1-µm-thick buried SiO_2 insulator layer and a 500-µm-thick intrinsic Si handle layer. The first step in fabricating the 3.5-mm-wide, 20-µm-thick membrane was to spin-on and pattern a layer of AZ9260 photoresist using UV lithography. The patterned photoresist layer acted as a mask for the deep reactive ion etching (DRIE) process. The buried SiO_2 insulator layer

acted as an etch-stop to prevent over-etching into the Si device layer. The insulator layer was then etched away using a BOE solution leaving only the 20-μm-thick membrane.

When spinning-on the AZ9260 photoresist, an additional drop of photoresist dripped onto the wafer near the end of the spin cycle. The extra drop left a thicker area of photoresist that was not uniformly spread on the wafer. This was not visually apparent and went unnoticed until after the DRIE etch process had begun. This resulted in portions of the membrane being slightly thicker than 20 μm such that the membrane was stiffer than expected. This effect is detailed further in Supplementary Note 3.

Bonding of the top and bottom Si substrates. The top and bottom Si substrates were bonded following the procedure described in Ref. 1 using an EVG 520 IS wafer bonder. The two substrates were precisely aligned using a custom-made alignment fixture. The fixture consisted of glass microscope slides bonded to a Si wafer. The fixture, along with the device, was placed inside the wafer bonder and subjected to a compressive force of 700 N at a temperature of 210°C for 60 minutes. This caused the SU-8 posts, located on the bottom Si substrate, to bond to the top Si substrate. Supplementary Fig. 1c shows a photograph of the device and alignment fixture inside the wafer bonder.

Supplementary Note 2. Calibration of the experimental setup.

Conduction heat transfer through borosilicate glass. The measurement method was first calibrated by measuring the thermal conductivity κ of a 1.1-mm-thick layer of borosilicate glass. Supplementary Fig. 2 shows a schematic of the experimental setup and the associated results. The near-field radiative heat transfer device was replaced with a 5×5 mm^2 layer of borosilicate

glass, and its thermal conductivity κ was retrieved by measuring the heat rate Q as a function of the temperature difference ΔT. Note that the cold side of the borosilicate layer, measured by a thermistor, was maintained at a constant temperature T_2 of 300 K in all experiments. Using Fourier's law and assuming one-dimensional conduction, a thermal conductivity κ of 1.0 W m^{-1} K^{-1} was experimentally determined. This value is in good agreement with published data for borosilicate glass[2].

Radiation heat transfer between Si surfaces separated by nanosize polystyrene spherical particles. In order to calibrate the measurement method for the case of radiation, the near-field radiative heat transfer device was replaced by two 5 × 5 mm^2, 525-μm-thick layers of intrinsic Si separated by vacuum gaps of 500 nm and 200 nm maintained by polystyrene spherical particles. This technique was used by Hu et al.[3] for measuring near-field radiative heat transfer between SiO_2 plates separated by a 1.6-μm-thick vacuum gap, since the polystyrene particles have a low thermal conductivity κ_p of 0.18 W m^{-1} K^{-1} and are essentially transparent in the infrared spectral band. A schematic of the setup and associated results are provided in Supplementary Fig. 3.

The Si emitter and receiver were cleaned in a UV/ozone chamber and were then rinsed in acetone, isopropanol and deionized water prior to depositing the polystyrene particles. The 500-nm-diameter particles were in a suspension of deionized water at a concentration of 1.15×10^{11} particles/mL. The suspension was sonicated with an Elmasonic Bath Sonicator to ensure a uniform distribution of particles. Since the particles were in a fairly high initial concentration, it was necessary to dilute them to ensure that only a monolayer of particles was deposited on the Si surface and to minimize conduction heat transfer between the emitter and receiver. The dilution was performed in two steps. First, 0.05 mL of the particle suspension was diluted with 100 mL of deionized water; second, 1 mL of the intermediate suspension from the first step was diluted with

115 mL of deionized water. The resulting concentration was 5×10^6 particles/mL. Using a syringe, 0.02 mL of the suspension was deposited on the Si receiver leaving approximately 10^4 polystyrene particles on the surface. The Si receiver was then dried on a hotplate before being aligned with the Si emitter and placed in the vacuum chamber. Preparation of the samples separated by 200-nm-diameter polystyrene particles was accomplished in a similar fashion. These particles were initially suspended in deionized water at a concentration of 1.8×10^{12} particles/mL and were diluted to a concentration of 7.8×10^6 particles/mL in two steps using sonication. A syringe was used to deposit 0.02 mL of the suspension on the surface of the Si receiver resulting in approximately 1.6×10^5 particles. According to the manufacturer specifications, the standard deviation in particle sizes was ±74 nm and ±30 nm for the 500 nm and 200 nm particles, respectively. This standard deviation was taken into account in the fluctuational electrodynamics simulations and is shown as colored bands in Supplementary Fig. 3b.

The heat flow through the sample Q ($\approx P_{HP}$) supplied by the thermoelectric (TE) heat pump is split into two contributions, namely the heat rate by radiation between the emitter and receiver $Q_{e\text{-}r}$, and the background heat rate Q_{back} (see Fig. 1c). Here, the background heat rate is solely due to conduction through the polystyrene particles. This background heat rate was determined by estimating the contact area between the particles and the Si surfaces via a Hertz model. Taking into account the force exerted by the masses of the TE heat pump, Si emitter, thermistor and copper (Cu) heat spreader, the contact area A between the 500 nm polystyrene particles and Si was determined to be 2241 nm^2. The background heat rate was thus estimated using Fourier's law:

$$Q_{\mathrm{back}} = N\frac{\kappa_{\mathrm{p}} A}{D}(T_{\mathrm{e}} - T_{\mathrm{r}}) \quad (1)$$

where N is the number of particles while D is the particle diameter which is the same as the gap size d separating the emitter and receiver. For a fixed receiver temperature of 300 K, the background heat rate was estimated to be 8.1×10^{-5} W and 9.7×10^{-4} W for temperature differences of 1 K and 120 K, respectively. Since the background heat transfer due to conduction through the polystyrene particles was always less than 1% of the heat rate by radiation, it was assumed that $Q \approx Q_{\mathrm{e\text{-}r}}$. The contact area between the 200 nm particles and the Si surfaces was estimated to be 194 nm^2 using the same process as described earlier, thus resulting in background heat rates of 2.7×10^{-5} W to 3.4×10^{-3} W for temperature differences of 1 K and 120 K, respectively. Although this heat rate was slightly larger than for the case of 500-nm-diameter particles, it was always less than 1.7% of the heat rate by radiation such that $Q \approx Q_{\mathrm{e\text{-}r}}$ was again assumed.

In general, there is a good agreement between experimental results and fluctuational electrodynamics predictions. For the case of 500 nm particles, the measured heat rate is 148.2×10^{-3} W at a temperature difference of 119.0 K. This differs from the predicted value of 143.5×10^{-3} W by 3.2%. At the lower end of the temperature difference, the experimentally measured heat rate is 5.3×10^{-3} W for a temperature difference of 4.6 K, which differs from the prediction of 3.7×10^{-3} W by 45.4%. However, the absolute value of the difference between the measured and experimental heat rates is 1.7×10^{-3} W, which is actually smaller than the difference at the high end of the temperature range of 4.6×10^{-3} W. For 200 nm particles, the measured heat rate is 227.7×10^{-3} W at a temperature difference of 108.9 K. This differs from the prediction of 205.1×10^{-3} W by 11.0%. At the lower end of the temperature difference, the experimentally measured heat rate is 24.5×10^{-3} W for a temperature difference of 14.7 K. This differs from the

prediction of 18.7×10^{-3} W by 31.3%. However, much like the case with 500 nm particles, the absolute value of the difference between the measured and experimental heat rates is 5.9×10^{-3} W which is less than the difference at the high end of the temperature range of 22.6×10^{-3} W. These discrepancies may be attributed to the difficulty in aligning the Si emitter and receiver. Unlike the near-field radiative heat transfer device, the Si emitter and receiver were aligned manually. Also, the imperfect dispersion of particles on the receiver and possible perturbations of the thermal near field by the polystyrene particles could have contributed to the discrepancies.

Supplementary Note 3. Membrane design.

As shown in Supplementary Fig. 1b, the device layer of an SOI wafer constitutes the membrane allowing the emitter to move relative to the receiver. The thickness of the device layer thus determined the thickness of the membrane. When designing the membrane, compliance and strength were considered. Since SOI wafers can be purchased with a variety of device layer thicknesses, a COMSOL model was created in order to determine an appropriate membrane thickness and width. Supplementary Fig. 4 shows the results for a 3.5-mm-wide, 20-μm-thick membrane. According to the model, a force of 4 mN is required to displace the emitter by 3.39 μm. At this deflection, the peak stress is 6.13 MPa and occurs at the edges where the membrane adjoins the bulk Si. The yield strength of Si is 7 GPa[4]; therefore, the membrane can flex from a nominal gap size of 3500 nm down to 150 nm without failing.

The expected gap size under a 4 mN load is predicted to be 110 nm. Since the SiO_2 stoppers have a height of 150 nm, a 4 mN applied force, corresponding to an added mass of 0.41 g, is theoretically sufficient to bring the device to a closed position. Additionally, according the

model, the effective spring coefficient, k, of the membrane is 1282 N m^{-1}. With a known spring coefficient, the gap size between the Si emitter and Si receiver can theoretically be determined based on the applied load.

Using the comprehensive heat transfer model to estimate the intermediate gaps between the closed and open position, it was found that a mass of 0.9 g results in a gap size of 1350^{+300}_{-200} nm, a mass of 1 g results in a gap size of 1050^{+150}_{-100} nm, a mass of 2 g results in a gap size of 600^{+100}_{-75} nm, a mass of 3 g results in a gap size of 350^{+75}_{-50} nm, and a mass of 4 g results in a gap size of 250^{+50}_{-25} nm. In practice, the mass required to bring the device in closed position was 5 g, corresponding to a force of 49.1 mN. The device had a stiffer membrane than expected which also flexed in a non-linear fashion. As mass was added to the device and the gap size decreased, the spring coefficient increased causing the membrane to become stiffer as it flexed. The effective spring coefficient k of the experimental device with an applied load of 1 g was calculated as approximately 3518 N m^{-1} while the spring coefficient was 14942 N m^{-1} with an applied load of 5 g. This may be due to the issue encountered during fabrication that was discussed in Supplementary Note 1. This resulted in the membrane being slightly thicker in certain areas causing it to be stiffer than designed.

SUPPLEMENTARY REFERENCES

1. Serra, S. G., Schneider, A., Malecki, K., Huq, S. E. & Brenner, W. A simple bonding process of SU-8 to glass to seal a microfluidic device. In *Proc. 3rd Int. Conf. on Multi-Material Micro Manufacture*, Borovets, Bulgaria, October 3-5 (2007).

2. Bansal, N.P. & Doremus, R.H. *Handbook of Glass Properties*, Academic Press, Orlando (1986).

3. Hu, L., Narayanaswamy, A., Chen, X. Y. & Chen, G. Near-field thermal radiation between two closely spaced glass plates exceeding Planck's blackbody radiation law. *Appl. Phys. Lett.* **92**, 133106 (2008).

4. Hsu, T.-R. *MEMS and Microsystems: Design, Manufacture, and Nanoscale Engineering*, Ch. 7, John Wiley and Sons, New Jersey (2008).
